# Molecular Variation in *AVP* and *AVPR1a* in New World Monkeys (Primates, Platyrrhini): Evolution and Implications for Social Monogamy

**DOI:** 10.1371/journal.pone.0111638

**Published:** 2014-10-31

**Authors:** Dongren Ren, Kelvin R. Chin, Jeffrey A. French

**Affiliations:** 1 Callitrichid Research Center, Department of Psychology, University of Nebraska at Omaha, Omaha, Nebraska, United States of America; 2 Key Laboratory for Animal Biotechnology of Jiangxi Province and the Ministry of Agriculture of China, Jiangxi Agricultural University, Nanchang, China; 3 Department of Biology, University of Nebraska at Omaha, Omaha, Nebraska, United States of America; University of Rouen, France

## Abstract

The neurohypophysial hormone arginine vasopressin (AVP) plays important roles in fluid regulation and vascular resistance. Differences in AVP receptor expression, particularly mediated through variation in the noncoding promoter region of the primary receptor for AVP (AVPR1a), may play a role in social phenotypes, particularly social monogamy, in rodents and humans. Among primates, social monogamy is rare, but is common among New World monkeys (NWM). AVP is a nonapeptide and generally conserved among eutherian mammals, although a recent paper demonstrated that some NWM species possess a novel form of the related neuropeptide hormone, oxytocin. We therefore characterized variation in the *AVP* and *AVPR1a* genes in 22 species representing every genus in the three major platyrrhine families (Cebidae, Atelidae and Pitheciidae). For *AVP*, a total of 16 synonymous substitutions were detected in 15 NWM species. No non-synonymous substitutions were noted, hence, *AVP* is conserved in NWM. By contrast, relative to the human *AVPR1a*, 66 predicted amino acids (AA) substitutions were identified in NWM. The AVPR1a *N*-terminus (ligand binding domain), third intracellular (G-protein binding domain), and *C*-terminus were variable among species. Complex evolution of *AVPR1a* is also apparent in NWM. A molecular phylogenetic tree inferred from *AVPR1a* coding sequences revealed some consensus taxonomic separation by families, but also a mixed group composed of genera from all three families. The overall d*N/*d*S* ratio of *AVPR1a* was 0.11, but signals of positive selection in distinct *AVPR1a* regions were observed, including the *N*-terminus, in which we identified six potential positive selection sites. AA substitutions at positions 241, 319, 399 and 409 occurred uniquely in marmosets and tamarins. Our results enhance the appreciation of genetic diversity in the mammalian *AVP/AVPR1a* system, and set the stage for molecular modeling of the neurohypophyseal hormones and social behavior in primates.

## Introduction

The neurohypophysial hormone arginine vasopressin (AVP) is synthesized primarily in the paraventricular nucleus (PVN) and supraoptic nucleus (SON) of the hypothalamus and then transported to the posterior pituitary for release into systemic circulation. Its two primary peripheral functions are related to osmoregulation and vasoconstriction [1], although AVP also exerts important regulatory effects in the central nervous system via neural projections that remain within the brain [2]. AVP is a nonapeptide, an ancient family of neuropeptides found in both vertebrates and invertebrates. All vertebrate nonapeptides, including AVP, are derived from arginine vasotocin (AVT) [3], [4]. The AVT gene was duplicated and subsequently modified to synthesize multiple oxytocin-like peptides in vertebrates. The evolution of vasopressin-like neuropeptides among vertebrates has been more conservative, with AVP replacing AVT in most mammals [3], [5], although pig, tenrec, opossum, and wallaby display variation in amino acid (AA) structure [6], [7]. Nevertheless, the ratio of nonsynonymous-to-synonymous divergence (*d*N/*d*S) of AVP is low across mammals (0.005), suggesting very strong conservation of this gene [6].

AVP exerts effects on multiple behavioral systems, including learning and memory, aggression, anxiety, affective states, and social behavior, primarily via interactions with the centrally expressed receptor subtype AVPR1a [2]. A causal role for AVP receptor polymorphisms and social behavior has focused on differences in repetitive microsatellite regions (short tandem repeats; STR) in the 5′ regulatory region of *AVPR1a*. In prairie voles, variation in the composition and length of these STRs are associated with species-level differences in gene expression *in vitro* and with differences in receptor expression and distribution in the brain [8]. These 5′ regulatory STRs also predict species differences in social monogamy, with some monogamous voles expressing STRs, and other non-monogamous vole species lacking these STR regions [9], [10]. In humans, there is a complex microsatellite (RS3) in the 5′ regulatory region of *AVPR1a* [11]. RS3 polymorphisms are associated with a variety of social phenotypes [12]–[18], including pair bonding associated behaviors [19].

However, broad comparative studies of *AVPR1a* STRs in rodents revealed that the causal links between promoter variation and sociality are not necessarily straightforward. Specifically, STRs are present in multiple species of non-monogamous voles, and are absent in only two non-monogamous species [12]. Further, the presence or absence of STR microsatellites does not account for social variation in other taxa, including *Peromyscus* [13] and primates [14]–[16].

Recently, attention has turned to variation in the coding regions for *AVPR1a*, previously considered to be unimportant for functional differences in either AVP signaling or social variation [9], [17], [18]. In 24 species representing the genus *Microtus*, exceptionally high variation in exon 1 of the *AVPR1a* gene has been demonstrated, particularly in the *N*-terminus ligand-binding extracellular and G-protein intracellular regions of the GPCR domain [19]. Similarly, a comparative study of *AVPR1a* coding regions in *Peromyscus* documented high rates of nonsynonymous nucleotide substitutions concentrated in the *N*-terminal extracellular domain within this taxon, with evidence of positive selection at these sites. However, no systematic AA substitutions were reliably associated with social monogamy among *Microtus* or *Peromyscu*s [13], [19], and the impact of coding variation in AVPR1a signaling activity appears to be minimal [13]. In New World monkeys (NWM), *AVPR1a* coding variation among four genera (*Aotus*, *Pithecia*, *Callicebus*, and *Saimiri*) confirms selective evolution of the *N*-terminus and G-protein binding regions in this taxon sufficient to clearly differentiate this clade from those containing other primates and mammals [16]. No unique signatures in either *AVPR1a* promoter or coding regions were identified that differentiated monogamous vs. non-monogamous NWM, although only one non-monogamous genus (*Saimiri*) was included in this study, and the sample was limited to only four of 17 genera that comprise the NWM.

In the present paper, we explore variation in the coding regions of *AVP/AVPR1a* across primates, with a particular focus on the entire clade of NWM. This taxon represents a particularly important test case for the link between AVP-signaling and sociality for two reasons. First, while social monogamy is rare among mammals and especially among primates [20], [21], this mating system is common in NWM, being present in more than 50% of genera (nine of 17) and 63.2% of species (74 of 117) in this group [21]. Secondly, a novel structural variant of the related neuropeptide oxytocin (OT) has been identified in four genera of NWM (*Callithrix, Cebus, Saimiri, Aotus*), in which a single in-frame mutation (Thy to Cyt) in the coding region of the *OT* gene results in an AA substitution from Leu to Pro at position 8 [22]. This suggests there may be unique selection for variability in neuropeptide signaling in this clade, given that both nonapeptides are derived from a duplication of the arginine vasotocin gene [23].

Because there are no comprehensive data regarding *AVP/AVPR1a* genetic variation throughout the NWM clade, we amplified, sequenced, and analyzed the *AVP* and *AVPR1a* coding region in species representing each of 17 genera in the three NWM families (Cebidae, Atelidae, and Pitheciidae). We examined variation in *AVPR1a* sequences across genera, in multiple species within some genera, and across multiple individuals within selected species. These NWM sequences were then contrasted with those from other primate taxa. Finally, we evaluated whether ligand and/or receptor nucleotide and subsequent AA substitutions were associated with social system variation in NWM.

## Materials and Methods

### Animals

A total of 22 NWM species were sampled, which covered all three families and eight subfamilies, and at least one species per genus. With the exception of *Cacajao*, we analyzed genomic DNA from at least two individuals, one male and one female, from each genus (sample range per species: 2–6). The species, DNA source, sex, and institutional source of each sample are presented in Table S1 in File S1. Sequences for *AVP* and *AVPR1a* for all other primates (hominoid, Old World, and prosimian primates) were accessed from UCSC Gene Browser/NCBI/Ensembl.

### Ethics Statement

All samples were accessed from archival blood or tissue banks, or from extracted DNA samples provided by the institutions listed in Table S1 in File S1. All institutions are licensed and/or accredited by appropriate agencies (e.g., USDA, AZA). IACUC information is also provided in Table S1 in File S1 where relevant.

### Amplification and Sequencing Gene Segments

Genomic DNA was extracted from whole blood or tissue samples using the DNeasy Blood and Tissue Kit (Qiagen) following the manufacturer’s protocol. DNA yield from the extractions was quantified using Nanodrop (Thermo Scientific) and DNA quality was measured using 0.8% agarose gel electrophoresis. The same sets of PCR primers were used to amplify *AVP* and *AVPR1a* coding regions in all NWM species (Table S2 in File S1). All primers were designed based on the *AVP* and *AVPR1a* conserved genomic regions in several taxa including human, chimpanzee and rhesus macaque (UCSC Genome Browser, http://genome.ucsc.edu/). All genomic regions were amplified under the following conditions: each 55 µL PCR reaction contained 100 ng genomic DNA, 4 mM MgCl_2_, 500 µM of each dNTP, 400 µM of each forward and reverse primers, 1.5 units of Taq DNA polymerase with 5.5 µL 10×PCR buffer. PCR was performed on a Techne 3 Prime Personal thermal cycler (Cole-Parmer) using a touchdown program with the following parameters: initial denaturation for 4 min at 95°C; followed by 12 cycles of 95°C for 35 s, 67°C (−0.6°C per cycle) for 35 s, and 72°C for 50 s; and followed by 23 cycles of 95°C for 35 s, 58°C for 35 s, and 72°C for 50 s; and a final extension at 72°C for 8 min. PCR products were run on 1% agarose gel, and only those with single bands were sequenced. PCR products were purified using QIAquick PCR Purification Kit (Qiagen), and sequenced directly in two directions with forward and reverse primers using an Applied Biosystems (ABI) 3730 48-capillary electrophoresis DNA analyzer, in the High-Throughput DNA Sequencing and Genotyping Core Facility at the University of Nebraska Medical Center.

### Sequence Alignment and Molecular Analysis

All coding sequences of *AVP* and *AVPR1a* generated in this study were deposited into GenBank (accession numbers: KJ641423 to KJ641466). Genomic sequences of *AVP* and *AVPR1a* in humans, nonhuman hominoid primates, Old World monkeys (OWM), and prosimians were acquired from UCSC Genome Bioinformatics. Sequence alignments of codon sequences were performed using MUSCLE in MEGA 6.0 [24]. We determined predicted AA structure for AVP and AVPR1a, and classified AA substitutions as conservative or radical according changes in polarity, charge, and volume: substitutions with a change in one or more categories were classified as radical, while substitutions with no changes in the three categories were classified as conservative [25].

Selection of models of nucleotide substitution was performed with MEGA 6.0 using a maximum likelihood statistical method [24], and the model with the lowest BIC score (Bayesian Information Criterion) was selected. A molecular phylogenetic tree of NWM was then generated using *AVPR1a* coding sequences with the Maximum Likelihood algorithm (1000 bootstrap, Tamura 3-parameter+G model) in MEGA 6.0 [24], with the prosimian *Microcebus AVPR1a* sequence identified as an outgroup relative to anthropoid primates. Positive selection on the entire *AVPR1a* gene was assessed using site models (M8/M8a), branch models (0/2), and branch-site models (A/B) with PAML 4.7 [26]. Evidence of positively selected extracellular, transmembrane, and intracellular regions in *AVPR1a* were estimated by using a sliding window analysis with a window length of 50 and a step size of 10 with the software DnaSP 5.10 to compare NWM species against those of humans and *Microcebus* [27].

### Classification of Social Monogamy

Social monogamy in mammals refers to a long-term or sequential living arrangement between an adult male and an adult female. This arrangement is frequently defined as: sharing the same territory, high rates of sociosexual behavior between pairmates, and often, but not always, biparental care. The presence or absence of genus-wide social monogamy in primates was assigned based on recent surveys [21], [28], and we note that our definition of social monogamy includes species that are not genetically monogamous [29].

## Results

### AVP diversity in primates

**Figure 1:**
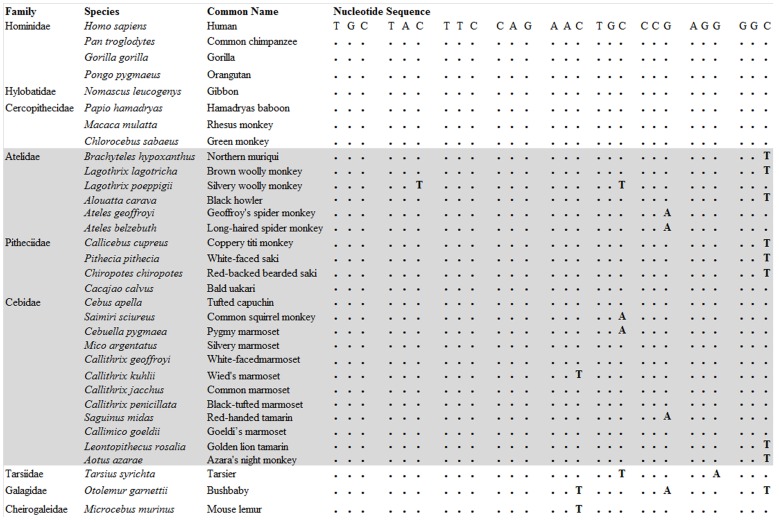
Arginine vasopressin (*AVP*) coding sequences for primates. New World monkeys (NWM) are indicated by shaded area. ‘**.**’ represents identity with the human *AVPR1a* sequence.

All primate species sampled had coding regions that yielded identical AA sequences for AVP (Cys-Tyr-Phe-Gln-Asn-Cys-Pro-Arg-Gly). In the families Hominidae, Hylobatidae, and Cercopithecidae, *AVP* coding sequences (27 nucleotides) were conserved, and no substitutions were identified (Figure 1). In NWM, *AVP* coding sequences were aligned among 22 species and contrasted with the human *AVP* nucleotide sequences. No non-synonymous substitutions were identified in this parvorder, and the nucleotides associated with AA at positions 1, 3, 4 and 8 were completely conserved across NWM. However, we identified a total of 16 synonymous substitutions in 15 species. The last nucleotide was more variable relative to other nucleotides, with a terminal T in 8 species. Nucleotides coding for the 6th AA exhibited two variants (TGT and TGA) in three genera from two families (Figure 1). Additionally, we noted species-specific nucleotide substitutions in cases in which more than one species per genus was sampled (*Callithrix* and *Lagothrix*). In tarsiers and two species of prosimian primates, six synonymous nucleotide substitutions were identified. *Otolemur* had synonymous substitutions at AA positions 5, 7 and 9, *Microcebus* had a single substitution at AA position 5, and *Tarsius* had two substitutions at positions 6 and 8 (Figure 1).

### AVPR1a diversity in primates

There were high levels of AA homology with human AVPR1a in hominoid primates and OWM. Nonhuman hominoid primates showed >98.4% sequence homology, and OWM exhibited >97.1% sequence homology. The prosimian *Microcebus* showed 89.5% homology with human AVPR1a, including a 9-amino acid deletion in the *N*-terminus. All nonhuman primates differed from human AVPR1a at positions 245 and 319. The amino acid at position 245 resides in the G-protein binding region, and constitutes a radical AA change compared with human. The amino acid at position 319 is located in the fourth extracellular region, and constitutes a radical AA change. Two substitution sites (positions 63 and 414) were unique to OWM.

Nucleotide substitutions in *AVPR1a* were widespread in NWM, with many substitutions resulting in AA changes throughout the coding region of this gene. Sequence homology with human *AVPR1a* among species in this taxon ranged from 92.3% (*Callithrix* and *Saguinus*) to 94.2% (*Chiropotes*). Relative to human *AVPR1a* sequences, we identified a total of 206 nucleotide substitutions in 22 species, which resulted in 66 predicted AA substitutions in the 418 AA that comprise AVPR1a (Figure S1 and S2 in File S1). Of the 66 AA substitutions, 10 were conserved across all NWM species (highlighted in green in Figure S1 in File S1)**.** Identical AA substitutions at eight positions occurred with high frequency (defined as >94%) across NWM genera (positions 3, 5, 25, 26, 43, 264, 321 and 337), and 27 of the substitutions were genus-specific (Figure S1 in File S1).

**Figure 2:**
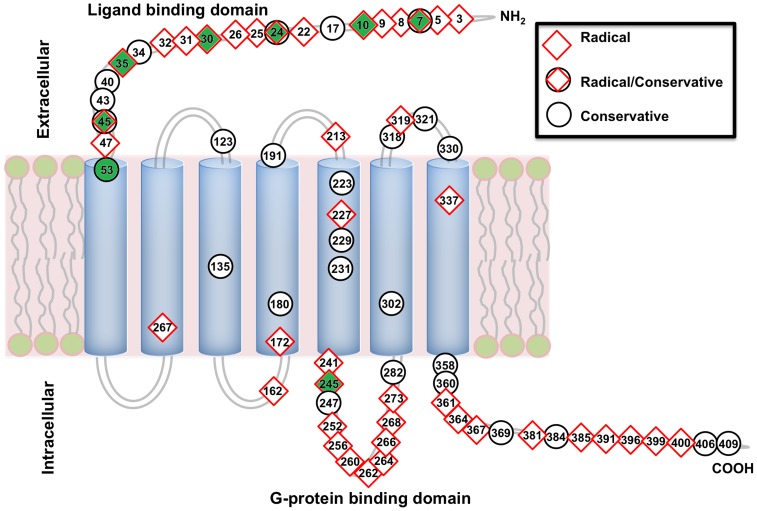
Structural model of AVPR1a. Numbers reflect amino acid substitutions in NWM relative to the human AVPR1a, and radical physicochemical substitutions are indicated by red diamonds and conservative changes by black circles. Potential positively selected sites are highlighted in green.

Across each region of AVPR1a for NWM, a higher percentage of AA substitutions were present in the extracellular *N*-terminus (20/52; 38.5%), the third intracellular region (13/54; 24.1%), and the intracellular *C*-terminus (15/67; 22.4%) than in other regions of AVPR1a (<22.2%) (Figure S2 in File S1). Transmembrane (TM) regions showed fewer substitutions compared with other regions (Figure S2 in File S1). A total of 47 AA substitutions in NWM AVPR1a involved radical changes, and 27 conservative changes (Figure 2; Table S3 in File S1). In three regions of the AVPR1a, multiple radical AA substitutions were noted across NWM. A total of 18, 13, and 9 radical physico-chemical substitutions were found in the *N*-terminus, the third intracellular region, and the *C*-terminus, respectively (Figure 2; Table S3 in File S1). Some AA substitutions had more than one change in physico-chemical category (Table S3 in File S1).

In three genera, we sampled and sequenced *AVPR1a* coding regions for more than one species. In *Callithrix* (*C. jacchus*, *C. kuhlii*, *C. geoffroyi*, and *C. penicillata*), the predicted AA for *AVPR1a* were identical across all four species. In two species sampled from the genus *Ateles*, we identified only one conservative AA substitution in the *N*-terminus (position 17). There was greater AVPR1a variation in the two species sampled from *Lagothrix*, with four AA changes being radical substitutions located in the *N*-terminus (positions 7, 10, 30 and 47), and the fifth was a conservative substitution located in the first TM region (position 53).

For four species in the genus *Callithrix*, we sampled 6 individuals per species to quantify intraspecific variation in nucleotide and AA sequences for AVPR1a. We identified only three synonymous nucleotide substitutions in the *AVPR1a* coding region among individuals in *C. kuhlii* and *C. penicillata* (AA positions 137, 151 and 302). No nucleotide substitutions were identified among individuals in *C. jacchus* or *C. geoffroyi*. All intraspecific nucleotide substitutions were located in exon 1. Thus, intraspecific individual variation in AVPR1a is low within the genus *Callithrix*.

### AVPR1a phylogeny and evolution in primates

**Figure 3:**
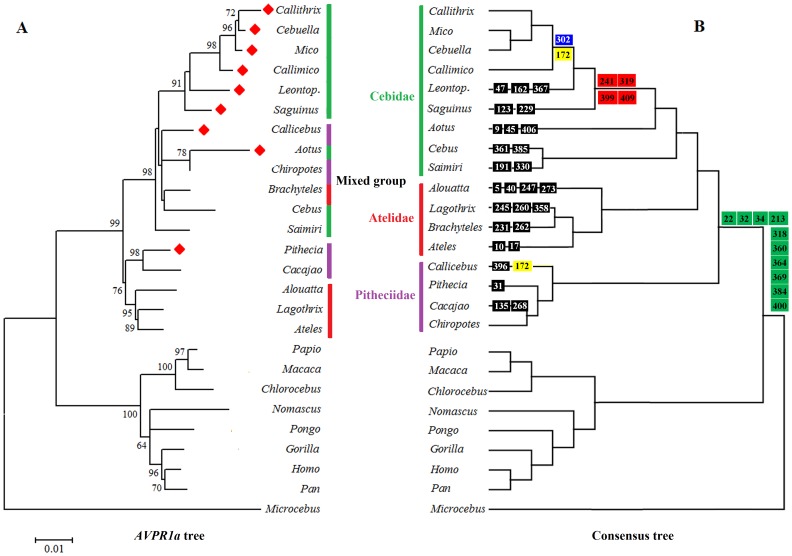
Molecular phylogenetic trees in primates. A. Tree inferred from *AVPR1a* nucleotide coding sequences in primates. If bootstrap support is <60, no value is shown at nodes. Scale bar indicates the branch length in nucleotide substitutions per site. A red diamond indicates a genus characterized by social monogamy. B. A consensus tree of primates based on 54 nuclear genes (34,927 bp; [30]). Families Cebidae, Atelidae and Pitheciidae are highlighted in green, red and purple lines, respectively. Amino acid substitutions of the *AVPR1a* gene are plotted on the consensus tree: NWM-specific (green square), Callitrichinae-specific (red square), marmoset-specific (blue square), marmoset and *Callicebus*-specific (yellow square), and genera-specific (black square).

A molecular phylogenetic tree was generated based on *AVPR1a* coding sequences (Figure 3A) and contrasted with a consensus molecular tree based on 54 nuclear genes ([30]; Figure 3B). Hominoid and OWM clearly clustered together, and were distinctly separate from the one species of prosimian for which the AVPR1a sequence was available. In NWM, all genera in the subfamily Calllitrichinae, and several genera in families Pitheciidae and Atelidae clustered together with high bootstrap support based on *AVPR1a* sequences. However, in the middle region of the tree, some genera from each family were intermixed, including *Cebus*, *Saimiri* and *Aotus* (Cebidae), *Brachyteles* (Atelidae), and *Callicebus* and *Chiropotes* (Pitheciidae), albeit with low bootstrap support (Figure 3A; bootstrap support with values <60 are not shown). Of the nine socially-monogamous NWM, six of nine genera clustered in one clade, with two additional genera in a sister clade, and *Pithecia* more distantly related.

**Figure 4:**
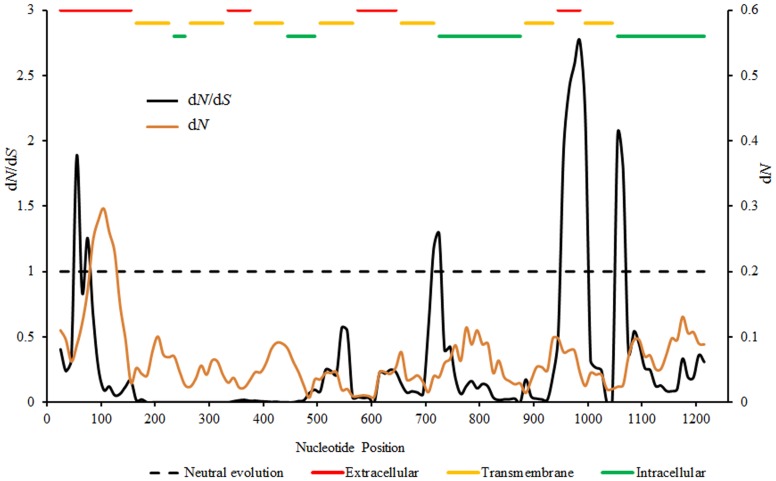
Sliding window analysis of d*N*/d*S* ratios and d*N* values along the NWM *AVPR1a*. The ratio/value are drawn over the midpoint window position (window length 50, step size 10) from whole coding region. A d*N*/d*S* ratio above 1 indicates possible positive selection in the region. The elements of *AVPR1a* are highlighted in red (extracellular), yellow (transmembrane), and green (intracellular) lines.

The overall *d*N/*d*S ratio for *AVPR1a* was 0.11, suggesting that purifying selection has acted upon *AVPR1a* in NWM. However, along the *AVPR1a* coding region against human *AVPR1a*, a sliding window analysis showed signals of positive selection (d*N*/d*S* >1.0) in multiple elements of the receptor, including the *N*-terminus, the third intracellular region, the fourth extracellular region, and the *C*-terminus (Figure 4). Other regions showed comparatively few non-synonymous mutations (Figure S2 in File S1), which led to low *d*N/*d*S ratios. PAML identified eight potential positive selection sites (*P*>0.05), including positions 7, 10, 24, 30, 35, 45, 53 and 245. In addition, sliding window analyses of NWM *AVPR1a* against *Microcebus* showed lower regional d*N*/d*S* ratios than those comparisons with humans (Figure S3 in File S1).

### AVPR1a diversity and social system

All genera within the subfamily Callitrichinae (marmosets and tamarins) are socially monogamous, and the species we analyzed exhibited four unique AA substitutions that differentiate this group from other NWM (Figure S1 in File S1). These substitutions include a radical change in an AA in the G-protein binding domain (Cys^241^), a radical change in the 4^th^ extracellular region (Asn^319^), and a radical and conservative change in the *C*-terminus (positions Val^399^ and Arg^409^, respectively). A conservative AA change in position Ile^302^ in the 6^th^ TM region was noted in marmosets only (*Callithrix*, *Cebuella*, *Mico*, and *Callimico*). These genera also shared an AA substitution with the monogamous *Callicebus* (a radical AA change at position 172 in the 4^th^ TM region; Gly^172^). Other genera-specific substitutions for the remaining monogamous genera (*Aotus*, *Pithecia*) can be found in Figure 3B. Thus, while there were no universal substitutions in AVPR1a AA sequences associated with social monogamy, we did identify unique AA substitutions in the Callitrichines, in which social monogamy characterizes the entire subfamily.

## Discussion

### AVP is conserved in primates

AVP is a neurohypophysial nonapeptide that is highly conserved in mammals [6]. Only two variants in eutherian AVP have been identified: Arg^8^-AVP is replaced by Lys^8^- AVP in pigs, opossum, wallabies [6], [7], and Glu^4^-AVP is replaced by Thr^4^-AVP in tenrecs [6]. Although a novel form of the related neuropeptide OT was recently identified in NWM [22], our broad survey of this taxon revealed no non-synonymous substitutions in the predicted AA sequence for AVP. Across primates, all species showed consensus amino acids for AVP. Thus, AVP ligand structure at the amino acid level in primates is consistent with that of most other eutherian mammals. We did note that NWM and prosimians possessed multiple synonymous mutations in the coding region for AVP, whereas nonhuman hominoid primates and OWM shared identical nucleotide sequences with humans. To the extent that AVP signaling systems constitute an important determinant of variation of sociality among primates [2], these effects are certainly not dependent on AVP ligand differences among species.

### Genetic variation in the AVPR1a gene

The nonhuman hominoid primates and OWM showed high homology with human in the 418 AA that comprise AVPR1a, which is consistent with their close phylogenetic relationship with humans. Two sites were unique to all nonhuman primates sampled (positions 245 and 319) and two were unique to OWM (positions 63 and 414), compared with human. Position 245 is located in the third intracellular region, suggesting possible differences in G-protein binding function in human vs. nonhuman primates. The single genus of prosimian primates characterized in our study (*Microcebus*) showed lower homology with human AVPR1a, which coincided with the greater phylogenetic genetic distance from humans.

NWM showed high rates of variability in the *AVPR1a* coding region (Figure S1 and S2 in File S1). Despite the AVP ligand being conserved in NWM, the major ligand recognition and binding region in the receptor (*N*-terminus) showed multiple radical physico-chemical substitutions. This observation is consistent with, and extends data from previous report showing that variable *N*-termini exist in the genus *Aotus* [16]. A short region (from Glu^37^ to Asn^47^) in the *N*-terminus of rat AVPR1a is necessary for AVP recognition and binding [31], [32]. In NWM, we documented substitutions in this specific region, including AA positions 43 and 45. It is not clear whether Lys^43^ or Glu^45^ play a role in AVP recognition and binding. The single residue Asn^47^ was conserved in primates with the exception of Asp^47^ in *Leontopithecus* and *Lagothrix* (Fig S2 in File S1). Both Glu^45^ and Asp^47^ constitute radical physico-chemical substitutions (Figure 2). Variable *N*-termini also were observed in the genus *Microtus* [19] and *Peromyscus* [13]. Whether this variation in *N*-terminus structure has functional significance is not clear, at least for *Peromyscus*, since signaling assays showed no detectable effect of the substitutions in this region on AVPR1a function [13]. Apart from signaling effects, tests of other functional consequences of *N*-terminus AA substitutions (e.g., recognition and binding activities between receptor and ligands) are needed to determine whether variability in NWM AVPR1a variants translate to functional differences.

The third intracellular region of AVPR1a plays important roles in signal transduction by binding G-protein [33]. This G-protein binding domain of AVPR1a was characterized by multiple radical substitutions in NWM (Figure 2). At position 256, *Callithrix* and *Alouatta* shared a unique substitution (Asn^256^). In this region, we also found an amino acid that appears to be unique to humans among all primates for which we had samples (Cys^245^). NWM and OWM showed different substitutions at position 264, both of which being radical changes. The radical changes found in this region might influence signal transduction, since the third intracellular region can lead to differences in receptor activation [34].

The *C*-terminus also showed variability in NWM, and multiple radical changes were identified. These findings indicated that these substitutions could alter AVPR1a function in NWM. Only five substitutions of 71 *C*-terminal residuals were observed across the genus *Peromyscus* [13], and hence *C*-terminus variation in *Peromyscus* is lower than in NWM (5/71 vs. 15/67 AA substitutions). No available data regarding *C*-terminus variation was reported in *Microtus* [19]. Since the *C*-terminus plays an important role in signal transduction [35], multiple substitutions in this region could influence the function of AVPR1a.

We assessed *AVPR1a* sequences in multiple species within three genera of NWM. Low levels of genetic variation were observed within genera, especially in the genus *Callithrix*. The highest rate of within-genus variation occurred in the contrast of two species of *Lagothrix* and two species of *Ateles*. More species per genera should be assessed to provide a more confident estimate of intrageneric variation in AVPR1a. Previous studies on species differences in *AVPR1a* coding region within single genera in rodents identified substantially higher levels of variation than in NWM. In 24 species in the genus *Microtus*, AVPR1a AA sequences contained over 100 substitutions, in addition to several deletions and insertions [19]. Within the genus *Peromyscus*, 27 substitutions were reported among eight species, including two insertions and one deletion [13]. The lower rate of AA substitutions in AVPR1a within single genera of NWM relative to rodents is consistent with the notion that evolution is more rapid in the rodent lineage, relative to the primate lineage [36].

We were able to assess individual polymorphisms in AVPR1a AA sequences in four species of *Callithrix*. There were no intraspecific differences in AA sequence in any of the four species. We identified only three synonymous nucleotide substitutions in exon 1 of *AVPR1a* in *C. kuhlii* and *C. penicillata*, indicating that AVPR1a is an evolutionarily conserved gene in genus *Callithrix*. Likewise, two studies on the coding region for human *AVPR1a* revealed only a synonymous substitution at position 136 in samples of 48 and 125 human participants, respectively [37], [38]. In *Peromyscus*, there was greater intraspecific variation, with individually-unique AA substitutions at one position identified in three species for which >4 individuals were sequenced [13]. Taken as a whole, therefore, within-species levels of polymorphism in AVPR1a coding regions are low, relative to levels of polymorphisms documented for promoter regions for this gene [14].

### AVPR1a evolution

In the phylogenetic tree derived from AVPR1a gene sequences, Hominoid primates, OWM, NWM and prosimians were separated according to conventional phylogenies with high bootstrap values. Three families in NWM generally clustered in a manner similar to the phylogenetic trees generated from 54 nuclear gene regions (Figure 3B) [30], mtDNA phylogenetic tree [39], and morphological characters tree [40]. Within the NWM, however, a mixed clade was observed. This mixed clade exhibited similar variants of *AVPR1a* in these six species, even though they belong to different families. Diverse traits are demonstrated in these six genera, including social monogamy in *Callicebus* and *Aotus*, and small body size in *Cebus*, *Saimiri* and *Aotus,* and different gestation lengths for each species [41]. Likewise, these genera live in diverse habitats ranging from wet topical forests to dry woodland habitats [42]. Thus, there is no consistent predictive variable (social system, body size, fluid regulation [43]) that can account for this cluster. This mixed group of genera in the AVPR1a phylogeny suggests a complexity in the evolution of this gene in NWM that has yet to be explained. The bootstrap values suggest two independent clades with social monogamy (*Callicebus*, *Aotus*, and the callitrichines; *Pithecia*), but further sampling of multiple species per genera, and additional details on the nature of the social phenotype among these taxa, is required to address this possibility.

Evidence for *AVPR1a* evolution was also indicated by quantification of d*N*/d*S* ratios, a sliding window analysis, and identification of potential positive selection sites. These results indicated that despite the low d*N*/d*S* for the entire *AVPR1a* gene, the ligand and G-protein binding regions may be under positive selection. The *C*-terminus is a critical domain for internalization and signal transduction [35], and d*N*/d*S* ratio of this domain indicated that this important element is underlying positive selection. In addition, in the fourth extracellular and the 7^th^ TM regions, though the regional d*N*/d*S* ratio also showed a higher value than 1.0, few substitutions were observed in the two regions (Figure S2 in File S1). In fact, selection could act on the synonymous sites [44]–[46]. Similar results were noted in the analysis of *N*-terminus AA substitutions in *Microtus* and *Peromyscus* [13], [19]. The d*N*/d*S* ratios again lead to the conclusion of complex of evolution of *AVPR1a* in NWM.

### AVPR1a variation and social monogamy

Social monogamy is generally rare among primates, but it is prevalent in NWM, with more than 50% of genera routinely displaying this social system [21], [28], [42], including all species in the subfamily Callitrichinae. We documented AA substitutions that are associated with some, but not all, monogamous NWM. Substitutions at six positions were noted in Callitrichine primates (marmoset and tamarin; or marmosets only), a taxon in which all species are socially monogamous. Four of the substitutions were radical physico-chemical substitutions, suggesting a possible functional change in AVPR1a. Whether these substitutions in AVPR1a contribute to monogamy in Callitrichine primates, or are simply a consequence of phylogeny, needs to be investigated further. Additionally, among the six substitutions listed above, only one was noted in another monogamous NWM genus (Gly^172^ in *Callicebus*; Figure S1 in File S1), but not in *Aotus* or *Pithecia*. While there are substitutions in AVPR1a associated with some monogamous NWM, the absence of a consistent pattern of substitutions uniquely associated with social monogamy suggests that if AVPR1a variation contributes to this social phenotype, it does so in complex ways. We note that the majority (six of nine genera) of socially-monogamous NWM clustered together with high bootstrap support in the phylogeny based on AVPR1a nucleotide sequences, suggesting that exploring the link between AVP signaling and social monogamy is worthy of additional attention.

Many of the substitutions in the social monogamous NWM genera were located in the *C*-terminus, and a region also characterized by a high d*N*/d*S* ratio. The *C*-terminus plays a critical role in internalization and signal transduction [35]. Human AVPR1a contains one proximal protein kinase C motif from position 382 to 384 (SRR). Deletion of this motif caused reduced receptor phosphorylation [47], and inhibited AVP stimulation of DNA synthesis and progression through the cell cycle [35]. We found that all NWM species display one specific substitution in this motif, Lys^384^. The substitution Arg^409^ occurs in the protein kinase *C* motif of marmosets and tamarins characterized by social monogamy. It would be fruitful to explore further whether variation in the *C*-terminus of AVPR1a contributes to altered receptor function and ultimately variation in social behavior.

Social monogamy is a complex social behavior. Recent hypotheses regarding the selective pressures leading to this trait include the difficulty of male defense of multiple females [21], protection from male infanticide [28], and certainty of paternity/genetic monogamy [29]. While the systematic evaluation of these hypotheses is beyond the scope of the present paper, it is worthwhile noting with regard to the third hypothesis that only one genera of NWM (*Aotus*) has been demonstrated to exhibit genetic monogamy, yet social monogamy and paternal care is common in NWM [29]. In any event, molecular sequence variation could reflect adaptation to these or other selective forces [16], [21], [28]. Research on *Peromyscus* suggests that mating system variation in rodents is not a consequence of simple genetic variation, and that social variation is likely to be mediated by multiple genetic mechanisms [13]. Candidate genes that may contribute to social monogamy include genes coding for OT, dopamine, and their respective cellular receptors [48], [49]. Evolutionary analyses in 25 species of *Microtus* showed that monogamy is not predicted by a single polymorphism in the promoter region of the *AVPR1a* gene [12]. In the present study, we documented six AA substitutions that were associated with social monogamy in some NWM. However, it is important to point out that these changes were specific to the subfamily Callitrichinae, and hence may represent simple phylogenetic differences and not functional variants vis-à-vis social system.

## Supporting Information

File S1 Contains Table S1, Sample information for the New World monkeys in this study. Table S2, PCR primers used to amplify genomic coding regions of AVP and AVPR1a (Underlined primer are the nested primers). Table S3, Radical or conservative change for each substitution in AVPR1a of NWM. N-term, *N*-terminus; TM, transmembrane region; IC, intracellular region; C-term, *C*-terminus. Reference sequence is human *AVPR1a*. Figure S1, Alignment of *AVPR1a* amino acid substitutions in NWM (shaded) and non-NWM relative to human *AVPR1a*. Green indicates NWM-specific substitutions, red represents unique substitutions in marmoset and tamarin, yellow indicates the substitution in marmoset and titi monkeys, blue indicates the marmoset-specific substitution, and ‘.’ represents identity with human. The vertical numbers in this figure indicate the amino acid position in AVRP 1a protein. *AVPR1a* sequences of *Tarsius syrichta* and *Otolemur garnettii* were not available from public data. The numbers of potential positive selection sites are framed. * indicates social monogamy. Figure S2, Alignment of the AVPR1a predicted amino acids in primates. The dot implicates the identity with human; pink indicates the extracellular regions; yellow represents the transmembrane regions; and blue indicates the intracellular regions. NWM are framed in the figure. Figure S3, Sliding window analysis of the d*N*/d*S* ratio and d*N* value along *AVPR1a* gene. A. *Microcebus* and 17 NWM genera. B. *Microcebus* and Hominoid. C. Old World monkeys against *Microcebus.* The ratio/value are drawn over the midpoint window position (window length 50, step size 10) from whole coding region. The elements of *AVPR1a* are highlighted in red (extracellular), yellow (transmembrane), and green (intracellular).
(DOCX)
