# Supplementary material for: Molecular Variation in AVP and AVPR1a in New World Monkeys (Primates, Platyrrhini): Evolution and Implications for Social Monogamy
Source: PLoS One. 2014 Oct 31;9(10):e111638. doi: 10.1371/journal.pone.0111638 (PMC4216101; doi:10.1371/journal.pone.0111638)
Supplement: File S1 — Contains Table S1, Sample information for the New World monkeys in this study. Table S2, PCR primers used to amplify genomic coding regions of AVP and AVPR1a (Underlined primer are the nested primers). Table S3, Radical or conservative change for each substitution in AVPR1a of NWM. N-term, N-terminus; TM, transmembrane region; IC, intracellular region; C-term, C-terminus. Reference sequence is human AVPR1a. Figure S1, Alignment of AVPR1a amino acid substitutions in NWM (shaded) and non-NWM relative to human AVPR1a. Green indicates NWM-specific substitutions, red represents unique substitutions in marmoset and tamarin, yellow indicates the substitution in marmoset and titi monkeys, blue indicates the marmoset-specific substitution, and ‘.’ represents identity with human. The vertical numbers in this figure indicate the amino acid position in AVRP 1a protein. AVPR1a sequences of Tarsius syrichta and Otolemur garnettii were not available from public data. The numbers of potential positive selection sites are framed. * indicates social monogamy. Figure S2, Alignment of the AVPR1a predicted amino acids in primates. The dot implicates the identity with human; pink indicates the extracellular regions; yellow represents the transmembrane regions; and blue indicates the intracellular regions. NWM are framed in the figure. Figure S3, Sliding window analysis of the dN/dS ratio and dN value along AVPR1a gene. A. Microcebus and 17 NWM genera. B. Microcebus and Hominoid. C. Old World monkeys against Microcebus. The ratio/value are drawn over the midpoint window position (window length 50, step size 10) from whole coding region. The elements of AVPR1a are highlighted in red (extracellular), yellow (transmembrane), and green (intracellular). (DOCX) [file pone.0111638.s001.docx]

**Molecular Variation in *AVP* and *AVPR1a* in New World Monkeys (Primates, Platyrrhini): Evolution and Implications for Social Monogamy**

Dongren Ren^1,2,3^, Kelvin Chin^1,3^, Jeffrey A. French^1,3^

^1^Callitrichid Research Center, Department of Psychology, University of Nebraska at Omaha, Omaha, NE 68182, U.S.A.

^2^Key Laboratory for Animal Biotechnology of Jiangxi Province and the Ministry of Agriculture of China, Jiangxi Agricultural University, Nanchang, 330045, China

^3^Department of Biology, University of Nebraska at Omaha, Omaha, NE 68182, U.S.A.

**Corresponding author:** Dongren Ren, PhD, Callitrichid Research Center, Department of Psychology, 6001 Dodge Street, University of Nebraska at Omaha, Omaha, NE 68182, U.S.A. Email: dren@unomaha.edu; Tel: (402) 554-3094; Fax: (402) 554-3121.

**Short title:** *AVP/AVPR1a* in New World monkeys

**Table S1. Sample information for the New World monkeys in this study.**

| Scientific name | Common name | Sex | DNA source^1^ | Institution | Provider | IACUC^2^ | |
| --- | --- | --- | --- | --- | --- | --- | --- |
| *Cebuella pygmaea* | Pygmy marmoset | 1M, 1F | DNA | Omaha's Henry Doorly Zoo | Edward Louis, Jr., Rick Brenneman, Douglas L Armstrong | Zoo tissue bank | |
| *Mico argentatus* | Silvery marmoset | 1M, 1F | Spleen | Bronx zoo, New York | D. McAloose, Jean A. Paré, Colleen McCann | Collected opportunistically at necropsy | |
| *Callithrix geoffroyi* | White-headed marmoset | 3M, 3F | Tail | University of Nebraska at Omaha | Jeffrey French, Heather A. Jensen | 12-099-12 | |
| *Callithrix kuhlii* | Wied's marmoset | 3M, 3F | Tail | University of Nebraska at Omaha | Jeffrey French, Heather A. Jensen | 12-099-12 | |
| *Callithrix jacchus* | Common marmoset | 3M, 3F | Tail | University of Nebraska at Omaha | Jeffrey French, Heather A. Jensen | 12-099-12 | |
| *Callithrix penicillata* | Black-tufted marmoset | 3M, 3F | Tail | University of Nebraska at Omaha | Jeffrey French, Heather A. Jensen | 12-099-12 | |
| *Callimico goeldii* | Goeldi’s marmoset | 2M, 2F | Whole blood | Chicago Brookfield Zoo | Mark Warneke, Jay Petersen | Zoo tissue bank | |
| *Leontopithecus rosalia* | Golden lion tamarin | 2M, 2F | Tail | University of Nebraska at Omaha | Jeffrey French, Heather A. Jensen | Tissue bank | |
| *Saguinus midas* | Red-handed tamarin | 1M, 1F | Whole  blood | Southwest National Primate Research Center | Jerilyn Pecotte | 1243SM | |
| *Cebus apella* | Tufted capuchin | 1M, 1F | Whole  blood | Alpha Genesis® Inc. | Melissa Ferguson | 11-007 | |
| *Saimiri sciureus* | Common squirrel monkey | 1M, 1F | DNA | Omaha's Henry Doorly Zoo | Edward Louis, Jr., Rick Brenneman, Douglas L Armstrong | Zoo tissue bank | |
| *Aotus azarae* | Azara's night monkey | 1M, 1F | Whole  blood | UT M. D. Anderson Cancer Center | Larry Williams, George W. Tustin, Brenda G. Webb | Tissue bank | |
| *Ateles geoffroyi* | Geoffroy's spider monkey | 1M, 1F | DNA | Omaha's Henry Doorly Zoo | Edward Louis, Jr., Rick Brenneman, Douglas L Armstrong | Zoo tissue bank | |
| *Ateles belzebuth* | Long-haired spider monkey | 1M, 1F | DNA | University of Texas at Austin | Anthony Di Fiore, Simone Loss | Tissue bank | |
| *Brachyteles hypoxanthus* | Northern muriqui | 2M, 2F | DNA;  Whole  blood | Muriqui Genetics Working Group | Karen B. Strier, Paulo B. Chaves, Sergio Lucena Mendes, Anthony Di Fiore, Valéria Fagundes,  Anthony Di Fiore, Simone Loss | Tissue bank | |
| *Lagothrix lagotricha* | Brown woolly monkey | 2M, 1F | DNA | Omaha's Henry Doorly Zoo; | Edward Louis, Jr., Rick Brenneman, Douglas L Armstrong; | Zoo tissue bank | |
|  |  |  | Liver | Louisville Zoo, Kentucky; | Elizabeth Rourk Hayden, Roy B. Burns, Steve Wing, | Approved by the Louisville Zoo Research Committee | |
| *Lagothrix poeppigii* | Silvery woolly monkey | 2M, 1F | DNA | Louisville Zoo, Kentucky; | Elizabeth Rourk Hayden, Roy B. Burns, Steve Wing; | Approved by the Louisville Zoo Research Committee | |
|  |  |  | Liver | University of Texas at Austin; | Anthony Di Fiore, Simone Loss | Tissue bank | |
| *Alouatta caraya* | Black howler | 1M, 1F | DNA | Omaha's Henry Doorly Zoo | Edward Louis, Jr., Rick Brenneman, Douglas L Armstrong | Zoo tissue bank | |
| *Callicebus cupreus* | Coppery titi monkey | 1M, 1F | Liver | California National Primate Research Center | Karen L. Bales, Tamara A.R. Weinstein | Approved by the UC-Davis IACUC |  |
| *Pithecia pithecia* | White-faced saki | 2M, 2F | DNA | Omaha's Henry Doorly Zoo; | Edward Louis, Jr., Rick Brenneman, Douglas L Armstrong; | Zoo tissue bank |  |
|  |  |  | Spleen | Bronx zoo, New York | D. McAloose, Jean A. Paré, Colleen McCann | Collected opportunistically at necropsy |  |
| *Chiropotes chiropotes* | [Red-backed bearded saki](http://en.wikipedia.org/wiki/Red-backed_Bearded_Saki) | 1M, 1F | DNA | Omaha's Henry Doorly Zoo | Edward Louis, Jr., Rick Brenneman, Douglas L Armstrong | Zoo tissue bank |  |
| *Cacajao calvus* | Bald uakari | 1M | DNA | University of Texas at Austin | Anthony Di Fiore, Simone Loss | Zoo tissue bank |  |

^1^DNA = extracted and purified DNA sample provided by institution; otherwise, we extracted DNA from the tissue source indicated.

^2^IACUC, Institutional Animal Care and Use Committee.

**Table S2. PCR primers used to amplify genomic coding regions of AVP and AVPR1a (Underlined primer are the nested primers).**

| Gene | Region | Forward primer (5'-3') | Reverse primer (5'-3') | Amplicon size (bp) |
| --- | --- | --- | --- | --- |
| AVP | Coding region | CCTGAATCACTGCTGACGGCT | CCTAAAGACTGCCATCACCCAT | 412 |
| AVPR1a | Coding region | CTTGAGTTGGGAACCCAGTGC | ATTGTTCACCTCGATCATGGA | 895 |
|  |  | CTTCTCGACTTCGCTAAAGTTG | CTACTAGCATGTAGGCCGATGC | 694 |
|  |  | TGGGACATCACCTACCGCTTC | AGAATGTCTGCGTGGGGAATC | 800 |
|  |  | TGGGACATCACCTACCGCTTC | CTACTGCCCTAGAAGATACGATG | 700 |
|  |  | ACATTCCCAGTCTCCCTGATT | TTGGTAGGAAGGTGAAAATGC | 599 |
|  |  | TTCCCAGTCTCCCTGATTGAT | CAGTCTTGTTCCCAATGAATTG | 564 |

Table S3. Radical or conservative change for each substitution in AVPR1a of NWM. N-term, *N*-terminus; TM, transmembrane region; IC, intracellular region; C-term, *C*-terminus. Reference sequence is human *AVPR1a*.

| **Element** | **Position** | **AA in human** | **AA in NWM** | **Substitution type** | **Proportion (%)** |
| --- | --- | --- | --- | --- | --- |
| N-term | 3 | L; Nonpolar and relatively small | F; Nonpolar and relatively large | Radical | 38.5 |
|  | 5 | A; Nonpolar | T: Polar | Radical |  |
|  | 7 | P; Neutral and small | L; Nonpolar and relatively small | Radical |  |
|  |  | P | A | Conservative |  |
|  | 8 | D; Negative | N; Neutral | Radical |  |
|  | 9 | A; Neutral, nonpolar, neutral and small | E; Negative, polar, polar and relatively small | Radical |  |
|  | 10 | G: Neutral, neutral and small | E; Negative, polar and relatively small | Radical |  |
|  |  | G: Neutral, neutral and small | R; Positive, polar and relatively large | Radical |  |
|  | 17 | P | A | Conservative |  |
|  | 22 | A; Neutral and small | V; Nonpolar and relatively small | Radical |  |
|  | 24 | G; Neutral, neutral and small | D; Negative, polar and relatively small | Radical |  |
|  |  | G | S | Conservative |  |
|  | 25 | A; Nonpolar | G; Polar | Radical |  |
|  | 26 | G; Polar | A; Nonpolar | Radical |  |
|  | 30 | R; Positive, polar and relatively large | Q; Neutral, polar and relatively small | Radical |  |
|  | 31 | E; Negative | Q; Neutral | Radical |  |
|  | 32 | A; Nonpolar | T: Polar | Radical |  |
|  | 34 | A | P | Conservative |  |
|  | 35 | L; Nonpolar and relatively small | F; Nonpolar and relatively large | Radical |  |
|  |  | L; Nonpolar, nonpolar and relatively small | S; Polar, neutral and small | Radical |  |
|  | 40 | G | S | Conservative |  |
|  | 43 | R | K | Conservative |  |
|  | 45 | V; Neutral, nonpolar, nonpolar and relatively small | E; Negative, polar, polar and relatively small | Radical |  |
|  |  | V | L | Conservative |  |
|  | 47 | N; Neutral | D; Negative | Radical |  |
| TM1 | 53 | L | V | Conservative | 4.2 |
| IC1 |  | - | - |  | 0 |
| TM2 |  | - | - |  | 0 |
| EC2 | 123 | L | M | Conservative | 6.7 |
| TM3 | 135 | M | I | Conservative | 4.5 |
| IC2 | 162 | Q; Neutral, polar and relatively small | R; Positive, polar and relatively large | Radical | 4.5 |
| TM4 | 172 | A; Nonpolar | G; Polar | Radical | 9.5 |
|  | 180 | V | L | Conservative |  |
| EC3 | 191 | M | V | Conservative | 7.1 |
|  | 213 | S; Polar | P; Nonpolar | Radical |  |
| TM5 | 223 | G | S | Conservative | 19 |
|  | 227 | A; Neutral and small | V; Nonpolar and relatively small | Radical |  |
|  | 229 | V | L | Conservative |  |
|  | 231 | I | M | Conservative |  |
| IC3 | 241 | Y; Nonpolar | C; Special | Radical | 24.1 |
|  | 245 | C; Neutral, special | R; Positive, polar and relatively large | Radical |  |
|  |  | C; Special | S; Neutral and small | Radical |  |
|  |  | C; Neutral, special | H; Positive, polar and relatively large | Radical |  |
|  | 247 | V | L | Conservative |  |
|  | 252 | A; Nonpolar | S; Polar | Radical |  |
|  | 256 | S; Neutral and small | N; Polar and relatively small | Radical |  |
|  | 260 | E; Negative, polar and relatively small | V; Neutral, nonpolar and relatively small | Radical |  |
|  | 262 | A; Nonpolar | G; Polar | Radical |  |
|  | 264 | V; Nopolar, nonpolar and relatively small | G; Polar, neutral and small | Radical |  |
|  | 266 | F; Nonpolar and relatively large | L; Nonpolar and relatively small | Radical |  |
|  | 267 | Q; Neutral, polar and relatively small | R; Positive, polar and relatively large | Radical |  |
|  | 268 | K; Positive, polar and relatively large | N; Neutral, polar and relatively small | Radical |  |
|  | 273 | A; Neutral and small | V; Nonpolar and relatively small | Radical |  |
|  | 282 | I | V | Conservative |  |
| TM6 | 302 | V | I | Conservative | 5 |
| EC4 | 318 | P | A | Conservative | 22.1 |
|  | 319 | M; Nonpolar, nonpolar and relatively small | N; Polar, polar and relatively small | Radical |  |
|  |  | M; Neutral, nonpolar, nonpolar and relatively small | K; Positive, polar, polar and relatively large | Radical |  |
|  | 321 | V | I | Conservative |  |
|  | 330 | I | V | Conservative |  |
| TM7 | 337 | G; Polar | A; Nonpolar | Radical | 5 |
| C-term | 358 | D | E | Conservative | 22.1 |
|  | 360 | V | I | Conservative |  |
|  | 361 | Q; Neutral, polar and relatively small | R; Positive, polar and relatively large | Radical |  |
|  | 364 | P; Neutral and small | L; Nonpolar and relatively small | Radical |  |
|  | 367 | Q; Neutral, polar and relatively small | H; Positive, polar and relatively large | Radical |  |
|  | 369 | M | L | Conservative |  |
|  | 381 | M; Nonpolar, nonpolar and relatively small | T; Polar, neutral and small | Radical |  |
|  | 384 | R | K | Conservative |  |
|  | 385 | Q; Polar, polar and relatively small | P; Nonpolar, neutral and small | Radical |  |
|  | 391 | N; Polar and relatively small | Y; Nonpolar and relatively large | Radical |  |
|  | 396 | N; Polar and relatively small | S; Neutral and small | Radical |  |
|  | 399 | G; Polar, neutral and small | V; Nonpolar, nonpolar and relatively small | Radical |  |
|  | 400 | M; Nonpolar, nonpolar and relatively small | T; Polar, neutral and small | Radical |  |
|  | 406 | K | R | Conservative |  |
|  | 409 | K | R | Conservative |  |


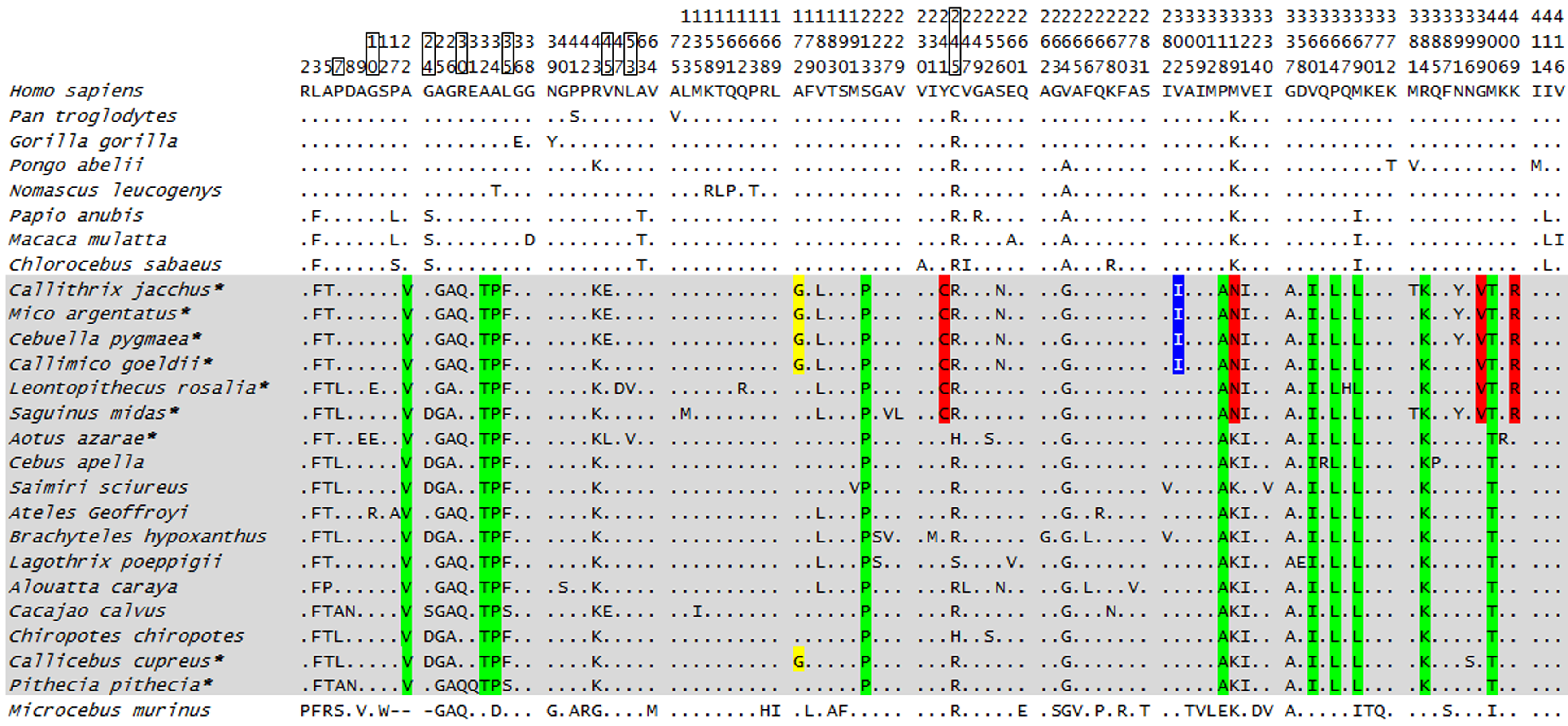


**Figure S1.** **Alignment of *AVPR1a* amino acid substitutions in NWM (shaded) and non-NWM relative to human *AVPR1a*.** Green indicates NWM-specific substitutions, red represents unique substitutions in marmoset and tamarin, yellow indicates the substitution in marmoset and titi monkeys, blue indicates the marmoset-specific substitution, and ‘.’ represents identity with human. The vertical numbers in this figure indicate the amino acid position in AVRP 1a protein. *AVPR1a* sequences of *Tarsius syrichta* and *Otolemur garnettii* were not available from public data. The numbers of potential positive selection sites are framed. * indicates social monogamy.


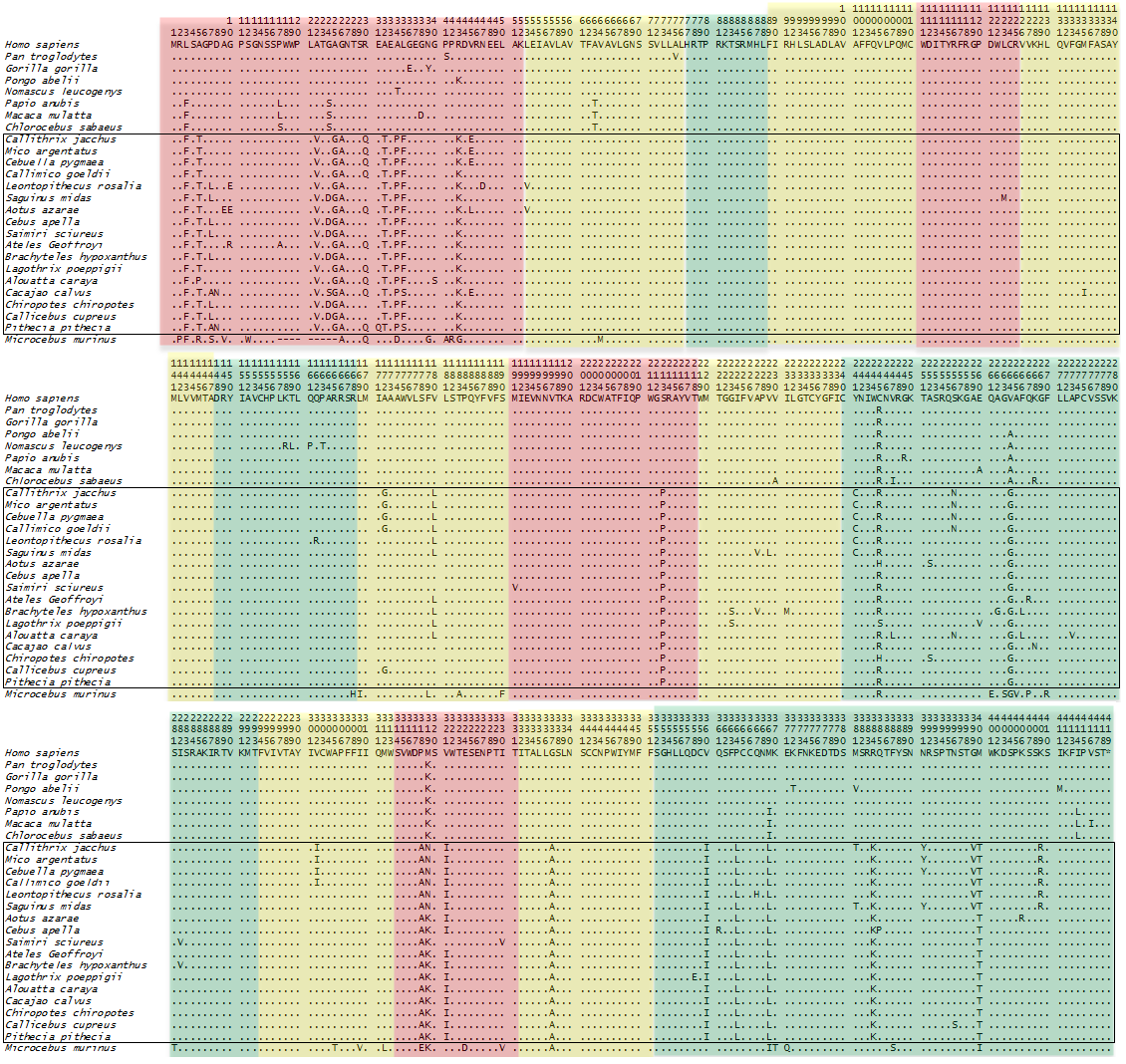


**Figure S2. Alignment of the AVPR1a predicted amino acids in primates.** The dot implicates the identity with human; pink indicates the extracellular regions; yellow represents the transmembrane regions; and blue indicates the intracellular regions. NWM are framed in the figure.


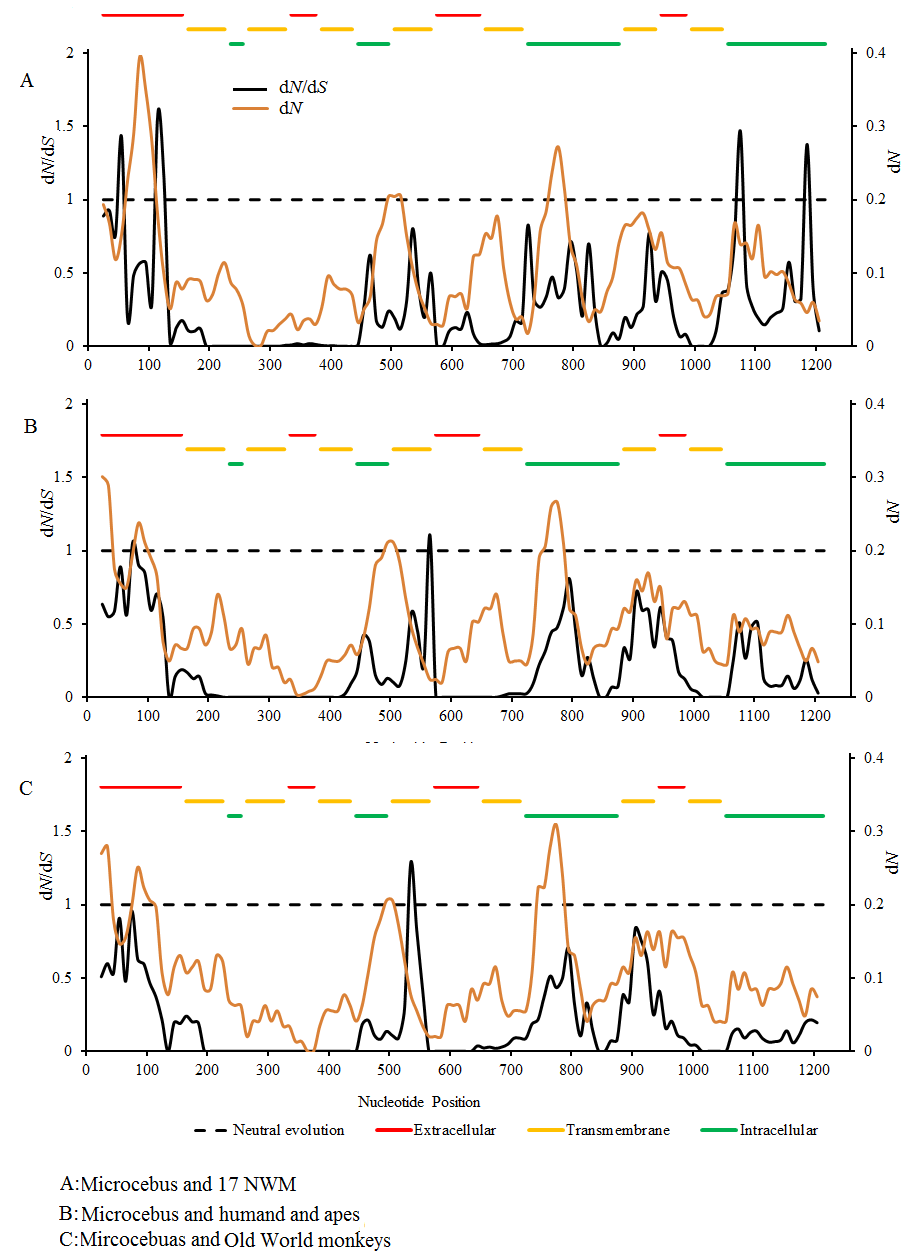


**Figure S3. Sliding window analysis of the d*N*/d*S* ratio and d*N* value along *AVPR1a* gene**. A. *Microcebus* and 17 NWM genera. B. *Microcebus* and Hominoid. C. Old World monkeys against *Microcebus.* The ratio/value are drawn over the midpoint window position (window length 50, step size 10) from whole coding region. The elements of *AVPR1a* are highlighted in red (extracellular), yellow (transmembrane), and green (intracellular).
